# Nomogram based on autophagy related genes for predicting the survival in melanoma

**DOI:** 10.1186/s12885-021-08928-9

**Published:** 2021-11-22

**Authors:** Guangtong Deng, Wenhua Wang, Yayun Li, Huiyan Sun, Xiang Chen, Furong Zeng

**Affiliations:** 1 The Department of Dermatology, Xiangya Hospital, Central South University, Changsha, Hunan China; 2 Hunan Key Laboratory of Skin Cancer and Psoriasis, Changsha, Hunan China; 3 Hunan Engineering Research Center of Skin Health and Disease, Changsha, 410008 Hunan China; 4 National Clinical Research Center for Geriatric Disorders, Xiangya Hospital, Central South University, Changsha, 410008 Hunan China

**Keywords:** Autophagy, Melanoma, Survival, Nomogram

## Abstract

**Background:**

Autophagy, a highly conserved lysosomal degradation pathway, is associated with the prognosis of melanoma. However, prognostic prediction models based on autophagy related genes (ARGs) have never been recognized in melanoma. In the present study, we aimed to establish a novel nomogram to predict the prognosis of melanoma based on ARGs signature and clinical parameters.

**Methods:**

Data from The Cancer Genome Atlas (TCGA) and the Genotype-Tissue Expression (GTEx) databases were extracted to identify the differentially expressed ARGs. Univariate, least absolute shrinkage and selection operator (LASSO) and multivariate analysis were used to select the prognostic ARGs. ARGs signature, age and stage were then enrolled to establish a nomogram to predict the survival probabilities of melanoma. The nomogram was evaluated by concordance index (C-index), receiver operating characteristic (ROC) curve and calibration curve. Decision curve analysis (DCA) was performed to assess the clinical benefits of the nomogram and TNM stage model. The nomogram was validated in GEO cohorts.

**Results:**

Five prognostic ARGs were selected to construct ARGs signature model and validated in the GEO cohort. Kaplan-Meier survival analysis suggested that patients in high-risk group had significantly worse overall survival than those in low-risk group in TCGA cohort (*P* = 5.859 × 10–9) and GEO cohort (*P* = 3.075 × 10–9). We then established and validated a novel promising prognostic nomogram through combining ARGs signature and clinical parameters. The C-index of the nomogram was 0.717 in TCGA training cohort and 0.738 in GEO validation cohort. TCGA/GEO-based ROC curve and decision curve analysis (DCA) demonstrated that the nomogram was better than traditional TNM staging system for melanoma prognosis.

**Conclusion:**

We firstly developed and validated an ARGs signature based-nomogram for individualized prognosis prediction in melanoma patients, which could assist with decision making for clinicians.

**Supplementary Information:**

The online version contains supplementary material available at 10.1186/s12885-021-08928-9.

## Introduction

Cutaneous melanoma (thereafter as “melanoma”) is one of the most aggressive skin malignancies, characterized by its high potential for invasiveness and metastasis, and limited response to treatment [1]. It is estimated that there is almost 287,723 new melanoma cases and 60,712 related deaths globally in 2018 [2]. Despite considerable improvement in the treatments for melanoma, there are still several factors contributing to the poor prognosis of melanoma, including delayed diagnosis and acquired resistance to targeted therapy and immunotherapy [3]. Prognostic prediction is necessary to help clinicians optimize therapeutic strategies. However, until now, the prognostic prediction still relies too much on the American Joint Committee on Cancer’s (AJCC) staging system for tumor-node-metastasis (TNM), which remains limitations because melanoma patients at the same stage vary widely in the survival outcomes [4, 5]. Therefore, it is imperative to elucidate prognostic predictors for melanoma.

Autophagy, a highly conserved lysosomal degradation pathway that supports nutrient recycling and metabolic adaptation, has been implicated as a double-edged sword in carcinogenesis [6]. In the pre-malignant cells, autophagy assists in sustaining physiological tissue homeostasis and avoids early-stage development of cancer through eradicating damaged organelles [7]. On the other hand, in established cancer, active autophagic flux provides energy and macromolecular precursors for tumor cell survival and growth even under harsh microenvironmental conditions [8]. Numerous studies have reported the involvement of autophagy in melanoma prognosis [9–12]. For example, down-regulated ATG5 contributed to tumorigenesis in the early-stage melanoma and were correlated with a reduced progression-free survival [13]. Also, Atg7 deficiency could prevent melanoma development by BrafV600E and allelic Pten loss and extend mouse survival [14]. These findings substantiate the close correlation between autophagy and melanoma, suggesting that autophagy-related genes (ARGs) are promising predictors for melanoma prognosis [15]. Considering that these studies mainly focused on assessing the function of one single gene, global expression patterns based on all the ARGs could increase the accuracy of prognostic prediction. To our best knowledge, no prognostic models have been established before based on multiple ARGs expression for melanoma.

Gene expression profiling and bioinformatics analysis have been used to explore the prognostic markers in many cancers. In our study, using these high-throughput expression data, we aimed to construct and validate a novel prognostic nomogram with more accuracy than traditional TNM staging system for melanoma patients through combining ARGs signature and clinical parameters.

## Materials and methods

### Data collection and processing

All the RNA sequencing data were extracted from The Cancer Genome Atlas (TCGA) dataset (https://portal.gdc.cancer.gov/), Gene Expression Omnibus (GEO) dataset (https://www.ncbi.nlm.nih. gov/geo/) and the Genotype-Tissue Expression (GTEx) project using the University of California Santa Cruz (UCSC) Xena website (https://xenabrowser.net/datapages/). The 222 ARGs were extracted from the Human Autophagy Database (HADb, http://www.autophagy.lu/project.html) [16]. TCGA data including 471 melanoma samples and 1 normal sample and GETx data including 812 normal tissue samples were used to identify the differentially expressed ARGs with log2 | fold-change (FC) | > 2 and adjusted *P*-value < 0.05 using R package “limma”. 460 melanoma samples in TCGA including overall survival were used for the following univariate and multivariate Cox analysis as the TCGA training cohort. 79 melanoma samples in GSE54467 (GEO validation cohort) were used for external validation. Before Cox analysis, the overlapped genes in TCGA and GEO cohorts were extracted and their expressions were normalized using “limma” and “sva” package in R.

### Functional annotation and pathway enrichment analysis

Gene ontology (GO), including biological process, cellular component and molecular function, and Kyoto Encyclopedia of Gene and Genomes (KEGG) [17, 18] pathway analysis were performed for these differentially expressed ARGs using “org. Hs.eg.db”, “clusterProfiler”, “enrichplot”, “ggplot2”, “GOplot” packages in R. Adjust *P* value less than 0.05 was considered statistically significant. GSEA (Gene Set Enrichment Analysis) was performed in java GSEA (verision 3.0) based on the Molecular Signatures Database version 6.233. With the 460 melanoma samples in TCGA dataset, KEGG and HALLMARK pathways, associated with high-risk and low-risk groups were identified by using hallmark gene sets and KEGG gene sets. FDR q value < 0.05, |NES| > 1 were considered statistically significant.

### Construction and evaluation of ARGs-based prognostic signature

Univariate Cox analysis was conducted to screen these differentially expressed ARGs related to overall survival in TCGA melanoma training cohort. Then, least absolute shrinkage and selection operator (LASSO) COX regression analysis and multivariate COX regression analysis were used to select the prognostic ARGs [19]. The optimal prognostic ARGs were determined to construct ARGs signature based on the Akaike information criteria (AIC). ARGs signature = β_1_ × expression of Gene_1_ + β_2_ × expression of Gene_2_ + ⋯ + β_n_ × expression of Gene_n_, where β is the corresponding coefficients generated by multivariate Cox analysis. The ARGs signature of each patient from the TCGA and GEO cohorts was calculated based on the above formula. The median signature from TCGA training cohort was regarded as the cutoff for both TCGA and GEO cohorts. All patients were strictly separated to high- and low-risk group with the cutoff. The survival difference for each cohort was evaluated by the Kaplan-Meier curve and log-rank test. Furthermore, to determine whether the ARGs signature could act as an independent prognostic factor, univariate and multivariate Cox analysis were performed in TCGA and GEO cohorts based on ARGs signature and clinicopathological factors including age, sex, body mass index (BMI), ulceration, Breslow depth and TNM stage. To further explore the predictive significance of ARGs signature in immunotherapy, GSE78220 dataset including 27 melanoma patients treated with anti-PD-1 therapy was used to divide high- and low-risk groups based on the same calculation formula and cutoff value described above. Fisher’s exact test was performed for low-risk and high-risk groups with and without responses to anti-PD-1 treatment. R package “glment”, “survminer” and “survival” were used in these analyses.

### Verification of the differential expression of prognostic ARGs

Gene expression profiling interactive analysis (GEPIA) has been used widely to explore the gene expression (http://gepia.cancer-pku.cn/index.html) between tumors and normal samples [20]. GSE15605 including 46 primary melanoma samples and 16 normal samples, and GSE46517 including 31 primary melanoma samples and 7 normal samples were used to validate the differential expression of prognostic ARGs.

### Establishment and validation of nomogram

ARGs signature, age and stage were enrolled to establish a nomogram in TCGA training cohort. The concordance index (C-index) and area under curve (AUC) in receiver operating characteristic (ROC) curve were generated to evaluate the discrimination of our nomogram. Calibration curve was plotted to measure the accuracy of the nomogram. Decision curve analysis (DCA) is widely used to evaluate the clinical value of models by integrating the preferences of the patients into analysis [21, 22]. DCA was performed to assess the clinical benefits of the nomogram and TNM stage model in both TCGA and GEO cohorts. The packages of R used in the analyses are as follows: “rms”, “foreign”, “survival”, and “stdca. R”.

## Results

### Screening of differentially expressed ARGs and enrichment analysis

**Fig. 1:**
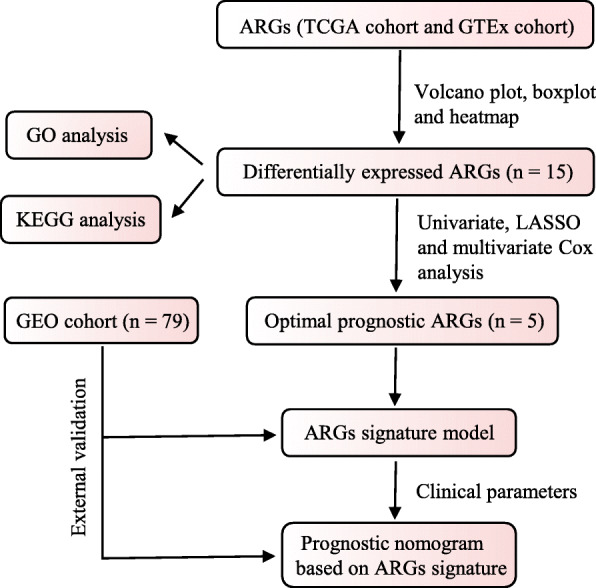
A flowchart of the study

**Fig. 2:**
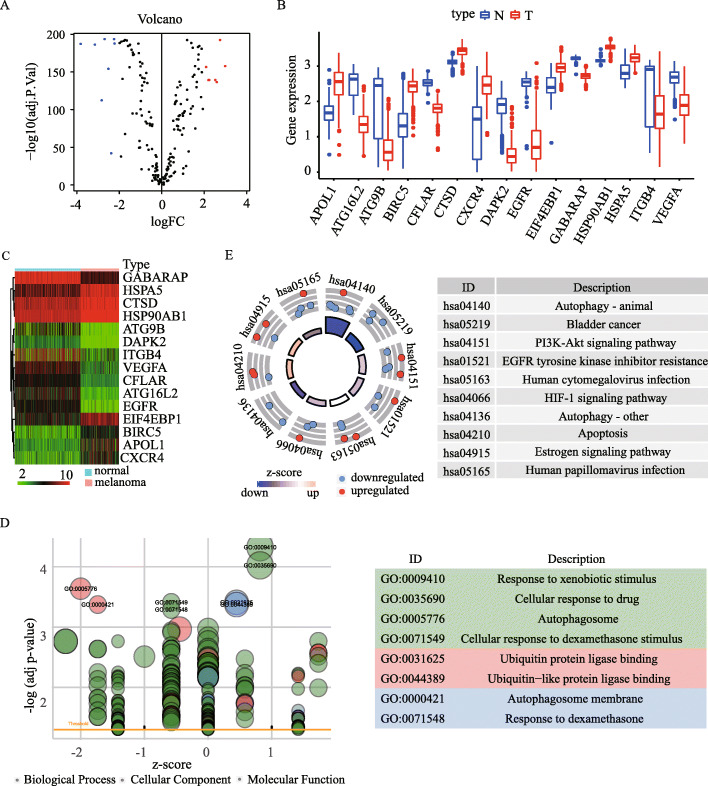
Screening of differentially expressed autophagy-related genes (ARGs) and enrichment analysis. (**A-C**) Volcano plot (**A**), boxplot (**B**) and heatmap (**C**) of differentially expressed ARGs between melanoma and normal samples in TCGA cohort with log2 | fold-change (FC) | > 2 and adjusted *P*-value < 0.05. (**D-E**) GO (**D**) and KEGG (www.kegg.jp/kegg/kegg1.html) pathway analysis (**E**)

The overall design and workflow for the study is presented in Fig. 1. Out of all ARGs, 15 differentially expressed ARGs were identified and visualized by volcano plot analysis (Fig. 2A). Boxplot and heatmap further demonstrated that seven ARGs have higher expression while eight ARGs have lower expression in melanoma than in normal skin (Fig. 2B and C). GO analysis was performed on these differentially expressed ARGs which mainly enriched in response to xenobiotic stimulus, ubiquitin protein ligase binding and autophagosome membrane (Fig. 2D). Moreover, KEGG pathway analysis revealed that these DE-ARGs were significantly enriched in autophagy, PI3K-Akt signaling pathway and HIF-1 signaling pathway (Fig. 2E).

### Identification of prognostic ARGs

**Fig. 3:**
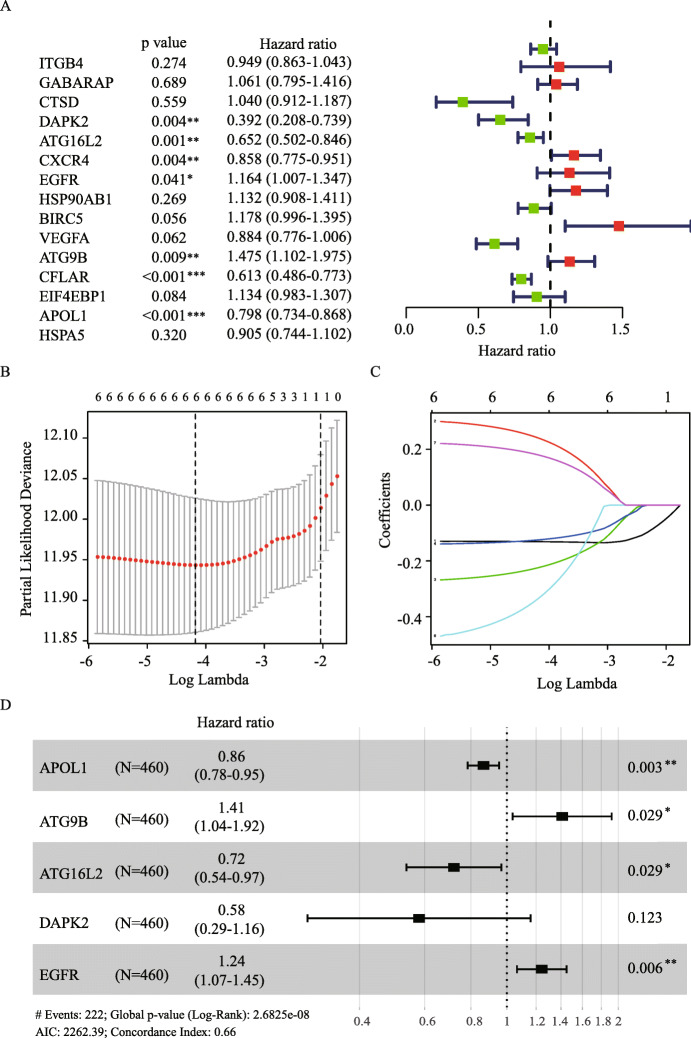
Identification of prognostic ARGs. (**A**) Univariate Cox analysis of 15 differentially expressed ARGs in TCGA cohort. (**B**) Selection of the optimal parameter (λ) in the LASSO model via 10-fold cross-validation in TCGA cohort. (**C**) LASSO coefficients produced by the regression analysis. (**D**) Multivariate Cox analysis of the candidate ARGs obtained from LASSO regression. *P* < 0.05 was regarded as statistically significant. *P < 0.05, **P < 0.01, ***P < 0.001

**Fig. 4:**
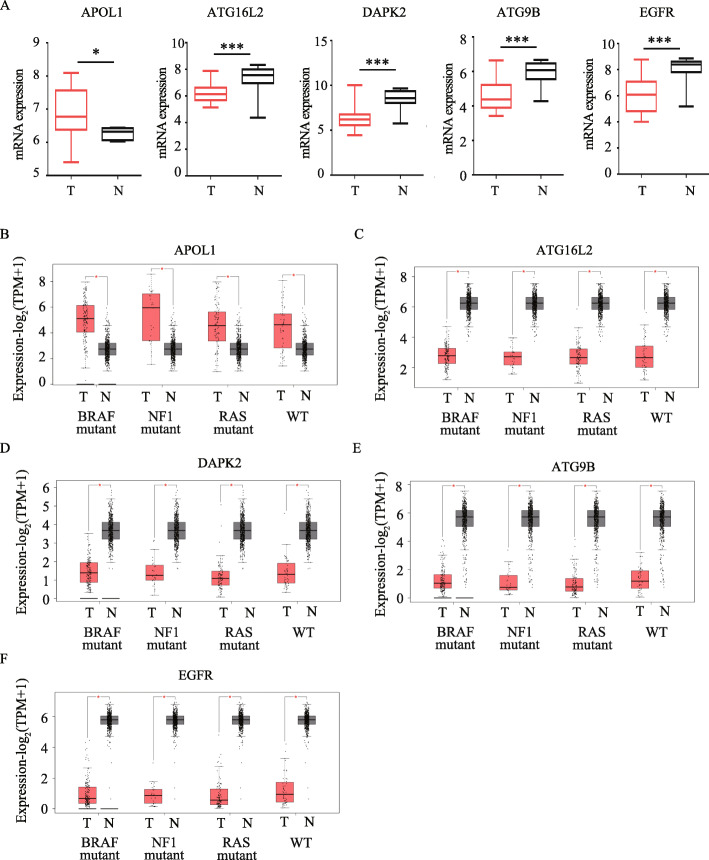
Verification of the differential expressions of prognostic ARGs. (**A**) The expression of APOL1 in GSE46517, and the expression of ATG16L2, DAPK2, ATG9B, and EGFR in GSE15605. *N* (T) = 31 and *N* (N) = 7 in GSE46517; *N* (T) = 46 and *N* (N) = 16 in GSE15605. *, *P* < 0.05; ***, *P* < 0.001. (**B-F**) The expression of APOL1 (**B**), ATG16L2 (**C**), DAPK2 (**D**), ATG9B (**E**) and EGFR (**F**) in three mutational signatures (BRAF, NF1 and RAS) and wild types (WT) of melanoma. The number of sorts: *N* (T) = 147 and *N* (N) = 558 in BRAF mutation; *N* (T) = 27 and *N* (N) = 558 in NF1 mutation; *N* (T) = 91and *N* (N) = 558 in RAS mutation; *N* (T) = 47and *N* (N) = 558 in WT. T = tumor, N = normal skin

Through univariate Cox analysis, we identified seven prognostic ATGs which have significant prognostic value (*P* < 0.05) (Fig. 3A). To avoid collinearity, we performed a LASSO logistic regression with tenfold cross-validation and finally six candidate ATGs were selected (Fig. 3B and C). Then, multivariable Cox analysis further showed that APOL1 (HR = 0.86, 95% CI: 0.78–0.95), ATG16L2 (HR = 0.72, 95% CI: 0.54–0.97), DAPK2 (HR = 0.58, 95% CI: 0.29–1.16) were considered as protective genes, while ATG9B (HR = 1.41, 95% CI: 1.04–1.92) and EGFR (HR = 1.24, 95% CI: 1.07–1.45) were risk genes for melanoma overall survival (Fig. 3D). To validate the differential expression of the above five genes, we analyze their expression in other GEO datasets. The results confirmed that APOL1 have higher expression in GSE46517 while ATG16L2, DAPK2, ATG9B and EGFR have lower expression in GSE15605 for primary melanoma compared with normal skin (Fig. 4A). Interestingly, these differential expressions were independent of the status of key melanoma mutations including BRAF, NF1, RAS mutations and triple wild type using GEPIA database (Fig. 4B-F).

### Construction and evaluation for ARGs signature model

**Fig. 5:**
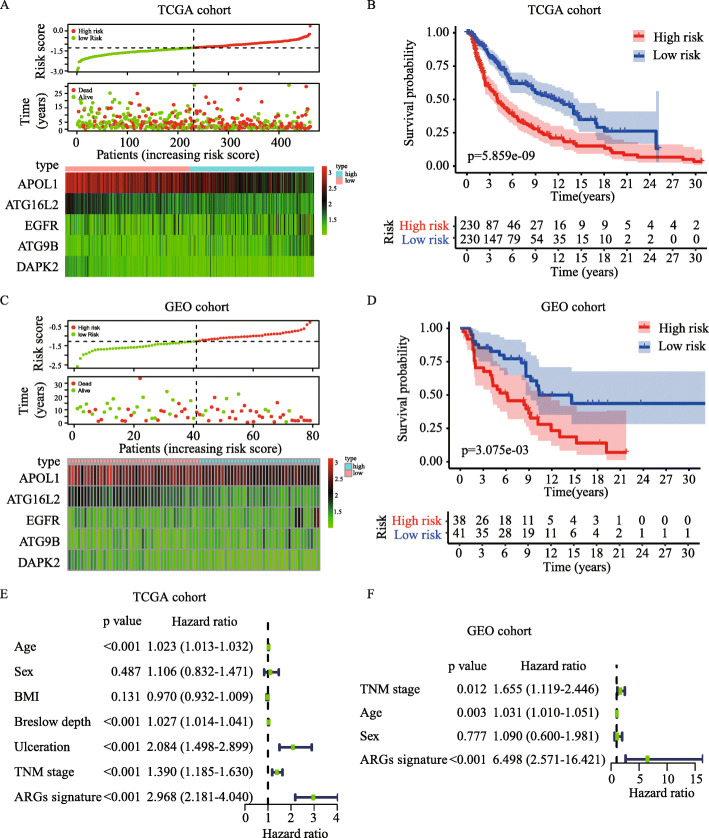
Construction and evaluation for ARGs signature model. **(A-D)** The risk score distribution, survival status and gene expression profiles in TCGA cohort (**A**) and GEO validation cohort (**C**). K-M survival curve of the ARGs signature for patients’ overall survival in the TCGA cohort (**B**) and GEO validation cohort (**D**). (**E-F**) Univariate Cox analysis of ARGs signature and clinical parameters in TCGA cohort (**E**) and GEO validation cohort (**F**)

ARGs signature model was established based on the regression coefficients of gene expression in multivariable Cox analysis with following formula: ARGs signature = (− 0.146 × expression of APOL1) + (0.344 × expression of ATG9B) + (− 0.329 × expression of ATG16L2) + (− 0.546 × expression of DAPK2) + (0.217 × expression of EGFR). The ARGs signature of each patient was calculated and all the patients were divided into high-risk (*n* = 230) and low-risk (*n =* 230) groups with the median risk score as the cutoff (− 1.270) in the TCGA training cohort. The patients’ survival time, life status, and ARGs expressions were shown in Fig. 5A. K-M survival analysis demonstrated that patients in high-risk group had significantly poorer overall survival than those in low-risk group (*P* = 5.859 × 10^− 9^) (Fig. 5B). Interestingly, disease free survival was also much shorter in the high-risk group (Fig. S1A-B). To verify the robustness of the ARGs signature model, the same formula and cutoff obtained from TCGA cohort was applied to GEO validation cohort. In line with our TCGA training cohort, patients in high-risk group (*n* = 38) had more death patients, poorer overall survival, increased risk gene expression and decreased protective gene expression than those in low-risk group (*n* = 41) in GEO cohort (Fig. 5C and D).

### Determination of ARGs signature as an independent prognostic factor

**Fig. 6:**
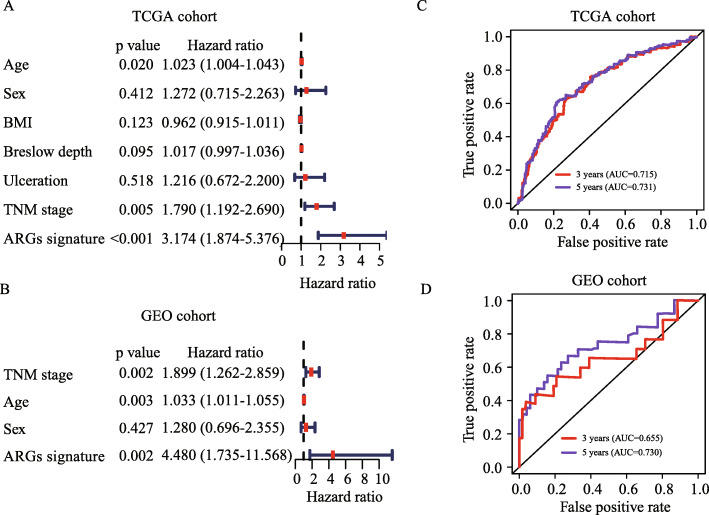
Prognostic performance of ARGs signature. (**A-B**) Multivariate Cox analysis of ARGs signature and clinical parameters in TCGA cohort (**A**) and GEO validation cohort (**B**). (**C-D**) ROC curve for predicting overall survival of 3-year (red) and 5-year (purple) in the TCGA cohort (**C**) and GEO validation cohort (**D**)

To evaluate whether the prognostic value of the ARGs signature was independent of clinical parameters, ARGs signature, age, sex, BMI, Breslow depth, ulceration and TNM stage were analyzed with univariate Cox analysis in TCGA cohort. The results demonstrated that ARGs signature (HR = 2.968, 95% CI: 2.181–4.040), age (HR = 1.023 95% CI: 1.013–1.032), Breslow depth (HR = 1.027, 95% CI: 1.014–1.041), ulceration (HR = 2.084, 95% CI: 1.498–2.899) and TNM stage (HR = 1.390, 95% CI: 1.185–1.630) were significantly associated with overall survival (Fig. 5E). In GSE validation cohort, only age, stage, sex and ARGs signature were available, in which ARGs signature (HR = 6.498, 95% CI: 2.571–16.421), age (HR = 1.031, 95% CI: 1.010–1.051) and TNM stage (HR = 1.655, 95% CI: 1.119–2.446) were also significantly associated with overall survival (Fig. 5F). Through multivariate Cox regression analysis, we found that the ARGs signature (HR = 3.174, 95% CI = 1.874–5.376), TNM stage (HR = 1.790, 95% CI = 1.192–2.690) and age (HR = 1.023, 95% CI = 1.004–1.043, *P* = 0.020) were independent prognostic predictors in TCGA cohort (Fig. 6A). These results were consistent in GEO cohort (Fig. 6B). To compare the predictive ability of our ARGs signature model, we plotted ROC curve. The ARGs signature model showed satisfactory predictive ability for 3- and 5-year overall survival rates, with AUC value of 0.715 and 0.731 respectively in TCGA cohort (Fig. 6C). In GEO cohort, the ARGs signature model also demonstrated a satisfactory predictive ability for the 3- and 5-year overall survival rates, with AUC value of 0.655 and 0.730 respectively (Fig. 6D).

To further explore the underlying mechanism of the prognostic ARGs signature, GSEA was conducted and suggested that 36 pathways in KEGG analysis were identified to be associated with low-risk group, mainly including apoptosis and immune activation-related pathways (Fig. S2A-C). Moreover, 11 pathways in hallmark gene sets were enriched in low-risk group including apoptosis, interferon alpha response, and interferon gamma response (Fig. S2D-F). The detailed results were shown in Table S2.

### Development and validation of a prognostic nomogram

**Fig. 7:**
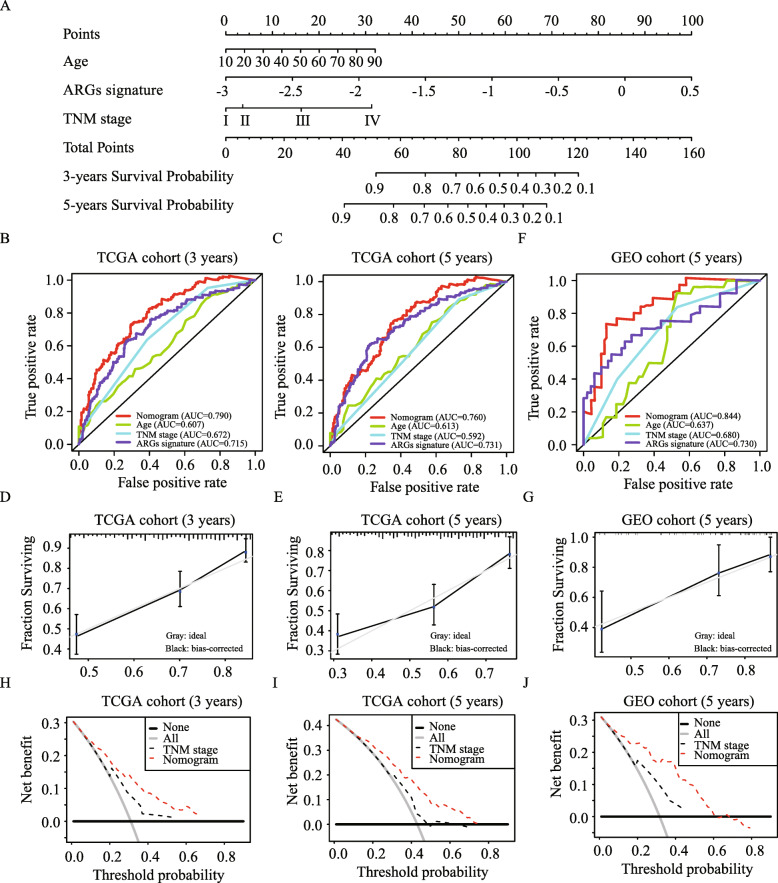
Development and validation of a prognostic nomogram based on ARGs signature. (**A**) Development the nomogram based on ARGs signature and independent clinical parameters. (**B-G**) The ROC curves for nomogram, age, stage and ARGs signature for predicting the overall survival at 3-year (**B**) and 5-year (**C**) in the TCGA cohort and 5-year (**F**) in the GEO validation cohort. The calibration curves of the nomogram for predicting overall survival at 3-year (**D**) and 5-year (**E**) in the TCGA cohort and 5-year in the GEO validation cohort (**G**). (**H-J**) Decision curve analysis of the nomogram and TNM stage system at 3-year (**H**) and 5-year (**I**) in the TCGA cohort and 5-year in the GEO validation cohort (**J**)

A prognostic nomogram based on the independent prognostic predictors including ARGs signature, age and TNM stage in TCGA training cohort were constructed to predict the overall survival of melanoma patients at 3 and 5 years (Fig. 7A). The C-index of the nomogram was 0.717 for predicting melanoma overall survival. The nomogram showed a better predictive ability for the 3- and 5-year overall survival rates with AUC values of 0.790 and 0.760 than ARGs signature (0.715 and 0.731), TNM stage system (0.672 and 0.592), or age (0.607 and 0.613) alone (Fig. 7B and C). Moreover, calibration plots showed excellent agreement between the prediction of our nomogram and actual prognosis for 3- and 5-year overall survival rates (Fig. 7D and E). Interestingly, the nomogram has more favorable predictive ability for the 5-year disease-free survival rates (Fig. S3A), with the C-index of 0.73 and AUC of 0.777. Besides, prediction and observation curve were consistent, and more clinic net benefit is added when using our nomogram (Fig. S3B and C). In addition, in GEO validation cohort, the C-index for the nomogram was 0.738. For the 5-year overall survival rates, the ROC curve demonstrated that the nomogram (AUC = 0.844) has more favorable predictive ability than other models (Fig. 7F); moreover, the calibration plot showed excellent agreement between prediction and observation curve (Fig. 7G). Furthermore, the DCA, for clinical usefulness evaluation, showed that the nomogram achieved the better net benefit than traditional TNM stage system in predicting the survival for melanoma patients for 3- and 5-year overall survival rates in TCGA and GEO validation cohort (Fig. 7H-J).

## Discussion

Autophagy is a highly-conserved dynamic process that deliver cellular proteins and damaged organelles to the lysosome for degradation. Recent studies showed that ARGs could regulate or be regulated by multiple signaling pathways such as PI3K/AKT/mTOR, P53/DRAM, RAS signaling pathway, which are essential for melanoma development and progression [23–27]. Therefore, ARGs are promising prognostic predictors in melanoma. In our study, we utilized high-throughput expression profiling of autophagy related genes to generate an ARGs signature model and a novel prognostic nomogram, which could guide individualized treatment for melanoma patients in high-risk group at the early stage.

In our study, we first screened out 15 differentially expressed ARGs based on TCGA and GETx databases and then confirmed five prognostic ARGs through univariate, lasso and multivariate Cox analysis. ApoL1, a BH3-only protein, is the major apoprotein of high-density lipoprotein and has never been studied in melanoma, but APOL1 is overexpressed in a variety of cancer cell types to induce autophagy and autophagy-associated cell death [28–30]. Liu et al. reported that APOL1 might be clinically relevant biomarkers for the diagnosis of pancreatic cancer [31]. Moreover, a recent study demonstrated that APOL1 could predict the prognosis of pancreatic cancer [32]. ATG16L2, a ubiquitously expressed homologue of ATG16L1, plays pivotal roles in autophagy pathway tumorigenesis by interacting with ATG5 [33]. ATG16L2 have been found to be prognostic marker for clear-cell renal cell carcinoma and stages I-III colon cancer [34, 35]. DAPK2 is a Ca^2+^-regulated serine/threonine kinase and phosphorylates mTORC1 through direct interaction, and promotes autophagy induction through suppressing mTOR activity [36]. In line with our finding, Li et al. reported that DAPK2 is a protective gene for melanoma prognosis [37]. As for ATG9B, which is located on chromosome 7 in humans, have been reported to be involved in the regulation of autophagy [38]. Studies have reported that ATG9B was overexpressed in clear-cell renal cell carcinoma but down-regulated in hepatocellular carcinoma and was associated with the cancer prognosis [35, 39]. Our study showed that increasing ATG9B predicted poor prognosis. EGFR is well studied in melanoma and has been found to activate autophagy and melanoma cell mobility [40, 41]. Another interesting finding is that HER3, a member of the EGFR family, is able to reactivate RAS-ERK signaling, allowing tumor cells to escape from the inhibitory effects of BRAF inhibitors [42]. That suggests that combination of EGFR and BRAF inhibitor shows synergistic effects in BRAF-mutant human melanoma in preclinical model [43]. All these studies supported our finding that EGFR is a risk gene for melanoma prognosis. In summary, these five genes may serve as the prognostic biomarkers and targets for melanoma therapy through modulating autophagy.

We next constructed and validated a novel AGRs signature model based on the five genes to predict the prognosis of melanoma patients. Compared with the TNM stage system, ARGs signature model showed satisfactory predictive ability of the 3- and 5-year overall survival rates, with higher AUC value. Patients with higher risk score had significantly poorer overall survival than those with lower risk score in both TCGA and GEO cohort. Moreover, the prognostic value of the ARGs signature was independent of clinical parameters through univariate and multivariate Cox analysis. That means ARGs signature is important for the prediction of melanoma prognosis.

Nowadays, immunotherapy has become a first-line therapy for metastatic melanoma patients. To further explore the potential significance of ARGs in immunotherapy, we analyzed the mRNA data and outcomes of 27 melanoma patients who received anti-PD-1 therapy in GSE78220 dataset. Using the same formula and cutoff, 9 and 18 patients were divided into high and low risk group. We found that only 2 (22.2%) high-risk patients responded to the immunotherapy, while 12 (66.7%) low-risk patients responded, suggesting that low-risk patients were more sensitive to the immunotherapy than the high-risk group (Fig. S4). Though the difference is statistically significant, further validation is required to draw convincing conclusions.

Lastly, we developed a prognostic nomogram based on the clinical parameters and ARGs signature. Nomogram has been widely used in oncology and medicine, which is a steady and credible tool through combining independent risk factors in a certain disease for their intuitive presentation [44–48]. Based on individual patients’ TNM stage, age and ARGs signature, our nomogram generates a numerical possibility for the overall survival. More importantly, this is the first nomogram to incorporate ARGs signature for the prediction of melanoma prognosis and has a better ability to predict the 3- and 5-year overall survival rates with higher AUC values than ARGs signature, TNM stage system or age alone. DCA is a well-established tool to evaluate the clinical value of models across a range of threshold probabilities to facilitate decisions about test selection and use [21, 22]. DCA also showed that our nomogram added more net benefit than traditional TNM stage system in melanoma prognosis prediction for 3- and 5-year overall survival rates. These findings suggested that our nomogram had a better predictive function in melanoma prognosis than TNM stage system.

Our nomogram is easy to be applied in clinic practice. Using our nomogram, clinician could give an accurate number for patient’s survival probability. For example, if the age, ARGs signature and TNM stage of a melanoma patient were 60 years old, − 2.0 and stage III, respectively, the corresponding points for age, ARGs signature and TNM stage were 32, 45 and 25 respectively. The total points value for this patient was 102. The 3-year and 5-year survival probability is about 36 and 20%, suggesting the poor prognosis in the patient. More precise individual treatment strategies should be taken for the patient including more aggressive treatment and closer follow-up.

Admittedly, our study had several limitations. First, the clinical characteristics from TCGA and GEO datasets were limited. Some information such as therapy and tumor pathological feature was not involved in our study. Second, an external validation based on prospective, multicenter, large-scale clinical trials was necessary to confirm the prediction ability of the nomogram. Finally, we used differentially expressed ARGs to construct the ARGs signature model and nomogram, which could leave out some ARGs with prognostic value but without difference in their expression between melanoma and normal skin.

## Conclusions

We detected and validated an ARGs signature model which could independently predict melanoma prognosis. Furthermore, we established and validated a novel prognostic nomogram with more accuracy than traditional TNM staging system for melanoma patients through combining both ARGs signature and clinical parameters.

## Supplementary Information

**Additional file 1: Figure S1.** Disease free survival between high and low risk group.

**Additional file 2: Figure S2.** Validation of a prognostic nomogram for disease free survival.

**Additional file 3: Figure S3.** GSEA analysis between high and low risk group in KEGG (www.kegg.jp/kegg/kegg1.html) and HALLMARK gene sets.

**Additional file 4: Figure S4.** Immunotherapy response in low- and high-risk patients by ARGs signature.

**Additional file 5: Table S1.** Clinical characteristics in TCGA and GEO datasets.

**Additional file 6: Table S2.** Enrichment for low-risk patients in KEGG and HALLMARK analysis.

## Data Availability

The data that support the findings of this study are openly available in TCGA dataset (https://portal.gdc.cancer.gov/), GEO dataset (GSE15605 and GSE46517) (https://www.ncbi.nlm.nih. gov/geo/) and the Genotype-Tissue Expression (GTEx) project using the University of California Santa Cruz (UCSC) Xena website (https://xenabrowser.net/datapages/).

## References

[1] Schadendorf D, van Akkooi ACJ, Berking C, Griewank KG, Gutzmer R, Hauschild A, Stang A, Roesch A, Ugurel S (2018). Melanoma. Lancet.

[2] Bray F, Ferlay J, Soerjomataram I, Siegel RL, Torre LA, Jemal A (2018). Global cancer statistics 2018: GLOBOCAN estimates of incidence and mortality worldwide for 36 cancers in 185 countries. CA Cancer J Clin.

[3] Arozarena I, Wellbrock C (2019). Phenotype plasticity as enabler of melanoma progression and therapy resistance. Nat Rev Cancer.

[4] Gershenwald JE, Scolyer RA, Hess KR, Sondak VK, Long GV, Ross MI, Lazar AJ, Faries MB, Kirkwood JM, McArthur GA, Haydu LE, Eggermont AMM, Flaherty KT, Balch CM, Thompson JF, for members of the American Joint Committee on Cancer Melanoma Expert Panel and the International Melanoma Database and Discovery Platform (2017). Melanoma staging: evidence-based changes in the American joint committee on Cancer eighth edition cancer staging manual. CA Cancer J Clin.

[5] Tian J, Yang Y, Li MY, Zhang Y (2020). A novel RNA sequencing-based prognostic nomogram to predict survival for patients with cutaneous melanoma: clinical trial/experimental study. Medicine (Baltimore).

[6] Amaravadi RK, Kimmelman AC, Debnath J (2019). Targeting autophagy in Cancer: recent advances and future directions. Cancer Discov.

[7] Rybstein MD, Bravo-San Pedro JM, Kroemer G, Galluzzi L (2018). The autophagic network and cancer. Nat Cell Biol.

[8] Levy JMM, Towers CG, Thorburn A (2017). Targeting autophagy in cancer. Nat Rev Cancer.

[9] Martin S, Dudek-Peric AM, Garg AD, Roose H, Demirsoy S, Van Eygen S, Mertens F, Vangheluwe P, Vankelecom H, Agostinis P (2017). An autophagy-driven pathway of ATP secretion supports the aggressive phenotype of BRAF(V600E) inhibitor-resistant metastatic melanoma cells. Autophagy.

[10] Noman MZ, Berchem G, Janji B (2018). Targeting autophagy blocks melanoma growth by bringing natural killer cells to the tumor battlefield. Autophagy.

[11] Ramkumar A, Murthy D, Raja DA, Singh A, Krishnan A, Khanna S, Vats A, Thukral L, Sharma P, Sivasubbu S, Rani R, Natarajan VT, Gokhale RS (2017). Classical autophagy proteins LC3B and ATG4B facilitate melanosome movement on cytoskeletal tracks. Autophagy.

[12] Wang L, Guo W, Ma J, Dai W, Liu L, Guo S, Chen J, Wang H, Yang Y, Yi X, Wang G, Gao T, Zhu G, Li C (2018). Aberrant SIRT6 expression contributes to melanoma growth: role of the autophagy paradox and IGF-AKT signaling. Autophagy.

[13] Liu H, He Z, von Rutte T, Yousefi S, Hunger RE, Simon HU (2013). Down-regulation of autophagy-related protein 5 (ATG5) contributes to the pathogenesis of early-stage cutaneous melanoma. Sci Transl Med.

[14] Xie X, Koh JY, Price S, White E, Mehnert JM (2015). Atg7 overcomes senescence and promotes growth of BrafV600E-driven melanoma. Cancer Discov.

[15] Li S, Song Y, Quach C, Guo H, Jang GB, Maazi H, Zhao S, Sands NA, Liu Q, In GK (2019). Transcriptional regulation of autophagy-lysosomal function in BRAF-driven melanoma progression and chemoresistance. Nat Commun.

[16] Wang Z, Gao L, Guo X, Feng C, Lian W, Deng K, Xing B (2019). Development and validation of a nomogram with an autophagy-related gene signature for predicting survival in patients with glioblastoma. Aging (Albany NY).

[17] Kanehisa M (2019). Toward understanding the origin and evolution of cellular organisms. Protein Sci.

[18] Kanehisa M, Furumichi M, Sato Y, Ishiguro-Watanabe M, Tanabe M (2021). KEGG: integrating viruses and cellular organisms. Nucleic Acids Res.

[19] Tibshirani R. The lasso method for variable selection in the cox model. Stat Med. 1997;16(4):385–95. 10.1002/(SICI)1097-0258(19970228)16:4<385::AID-SIM380>3.0.CO;2-3. 10.1002/(sici)1097-0258(19970228)16:4<385::aid-sim380>3.0.co;2-3 9044528

[20] Tang Z, Li C, Kang B, Gao G, Li C, Zhang Z (2017). GEPIA: a web server for cancer and normal gene expression profiling and interactive analyses. Nucleic Acids Res.

[21] Fitzgerald M, Saville BR, Lewis RJ (2015). Decision curve analysis. JAMA.

[22] Zhang Z, Rousson V, Lee WC, Ferdynus C, Chen M, Qian X (2018). Guo Y, written on behalf of AMEB-DCTCG: decision curve analysis: a technical note. Ann Transl Med.

[23] Dou Z, Pan JA, Dbouk HA, Ballou LM, DeLeon JL, Fan Y, Chen JS, Liang Z, Li G, Backer JM (2013). Class IA PI3K p110beta subunit promotes autophagy through Rab5 small GTPase in response to growth factor limitation. Mol Cell.

[24] Iershov A, Nemazanyy I, Alkhoury C, Girard M, Barth E, Cagnard N, Montagner A, Chretien D, Rugarli EI, Guillou H, Pende M, Panasyuk G (2019). The class 3 PI3K coordinates autophagy and mitochondrial lipid catabolism by controlling nuclear receptor PPARalpha. Nat Commun.

[25] Robin M, Issa AR, Santos CC, Napoletano F, Petitgas C, Chatelain G, Ruby M, Walter L, Birman S, Domingos PM, Calvi BR, Mollereau B (2019). Drosophila p53 integrates the antagonism between autophagy and apoptosis in response to stress. Autophagy.

[26] White E (2016). Autophagy and p53. Cold Spring Harb Perspect Med.

[27] Kinsey CG, Camolotto SA, Boespflug AM, Guillen KP, Foth M, Truong A, Schuman SS, Shea JE, Seipp MT, Yap JT, Burrell LD, Lum DH, Whisenant JR, Gilcrease GW, Cavalieri CC, Rehbein KM, Cutler SL, Affolter KE, Welm AL, Welm BE, Scaife CL, Snyder EL, McMahon M (2019). Publisher correction: protective autophagy elicited by RAF-->MEK-->ERK inhibition suggests a treatment strategy for RAS-driven cancers. Nat Med.

[28] Chidiac M, Fayyad-Kazan M, Daher J, Poelvoorde P, Bar I, Maenhaut C, Delree P, Badran B, Vanhamme L (2016). ApolipoproteinL1 is expressed in papillary thyroid carcinomas. Pathol Res Pract.

[29] Hu CA, Klopfer EI, Ray PE (2012). Human apolipoprotein L1 (ApoL1) in cancer and chronic kidney disease. FEBS Lett.

[30] Jian L, Yang G (2020). Identification of key genes involved in diabetic peripheral neuropathy progression and associated with pancreatic Cancer. Diabetes Metab Syndr Obes.

[31] Liu X, Zheng W, Wang W, Shen H, Liu L, Lou W, Wang X, Yang P (2017). A new panel of pancreatic cancer biomarkers discovered using a mass spectrometry-based pipeline. Br J Cancer.

[32] Sharpnack MF, Chen B, Aran D, Kosti I, Sharpnack DD, Carbone DP, Mallick P, Huang K (2018). Global transcriptome analysis of RNA abundance regulation by ADAR in lung adenocarcinoma. EBioMedicine.

[33] Wen J, Liu H, Wang L, Wang X, Gu N, Liu Z, Xu T, Gomez DR, Komaki R, Liao Z, Wei Q (2018). Potentially functional variants of ATG16L2 predict radiation pneumonitis and outcomes in patients with non-small cell lung Cancer after definitive radiotherapy. J Thorac Oncol.

[34] Mo S, Dai W, Xiang W, Li Y, Feng Y, Zhang L, Li Q, Cai G (2019). Prognostic and predictive value of an autophagy-related signature for early relapse in stages I-III colon cancer. Carcinogenesis.

[35] Wan B, Liu B, Yu G, Huang Y, Lv C (2019). Differentially expressed autophagy-related genes are potential prognostic and diagnostic biomarkers in clear-cell renal cell carcinoma. Aging (Albany NY).

[36] Boroughs LK, DeBerardinis RJ (2015). Metabolic pathways promoting cancer cell survival and growth. Nat Cell Biol.

[37] Li Q, Zhang LY, Wu S, Huang C, Liu J, Wang P, Cao Y (2019). Bioinformatics analysis identifies MicroRNAs and target genes associated with prognosis in patients with melanoma. Med Sci Monit.

[38] Yamada T, Carson AR, Caniggia I, Umebayashi K, Yoshimori T, Nakabayashi K, Scherer SW (2005). Endothelial nitric-oxide synthase antisense (NOS3AS) gene encodes an autophagy-related protein (APG9-like2) highly expressed in trophoblast. J Biol Chem.

[39] Wang N, Tan HY, Li S, Feng Y (2017). Atg9b deficiency suppresses autophagy and potentiates endoplasmic reticulum stress-associated hepatocyte apoptosis in Hepatocarcinogenesis. Theranostics.

[40] Katunaric M, Zamolo G, Jonjic N (2015). EGFR activated cell mobility - a link to melanoma ulceration. Med Hypotheses.

[41] Senos Demarco R, Uyemura BS, Jones DL (2020). EGFR signaling stimulates autophagy to regulate stem cell maintenance and lipid homeostasis in the Drosophila testis. Cell Rep.

[42] Girotti MR, Marais R (2013). Deja vu: EGF receptors drive resistance to BRAF inhibitors. Cancer Discov.

[43] Kenessey I, Kramer Z, Istvan L, Cserepes MT, Garay T, Hegedus B, Dobos J, Timar J, Tovari J (2018). Inhibition of epidermal growth factor receptor improves antitumor efficacy of vemurafenib in BRAF-mutant human melanoma in preclinical model. Melanoma Res.

[44] Balachandran VP, Gonen M, Smith JJ, DeMatteo RP (2015). Nomograms in oncology: more than meets the eye. Lancet Oncol.

[45] Chen L, Zeng F, Yao L, Fang T, Liao M, Long J, Xiao L, Deng G (2020). Nomogram based on inflammatory indices for differentiating intrahepatic cholangiocarcinoma from hepatocellular carcinoma. Cancer Med.

[46] Deng G, Yao L, Zeng F, Xiao L, Wang Z (2019). Nomogram for preoperative prediction of microvascular invasion risk In hepatocellular carcinoma. Cancer Manag Res.

[47] Harrell FE Jr, Lee KL, Mark DB. Multivariable prognostic models: issues in developing models, evaluating assumptions and adequacy, and measuring and reducing errors. Stat Med. 1996;15(4):361–87. 10.1002/(SICI)1097-0258(19960229)15:4<361::AID-SIM168>3.0.CO;2-4. 10.1002/(SICI)1097-0258(19960229)15:4<361::AID-SIM168>3.0.CO;2-4 8668867

[48] Wang Y, Zhao W, Xiao Z, Guan G, Liu X, Zhuang M (2020). A risk signature with four autophagy-related genes for predicting survival of glioblastoma multiforme. J Cell Mol Med.

