# Supplementary figures and images for: Nomogram based on autophagy related genes for predicting the survival in melanoma
Source: BMC Cancer. 2021 Nov 22;21:1258. doi: 10.1186/s12885-021-08928-9 (PMC8607622; doi:10.1186/s12885-021-08928-9)

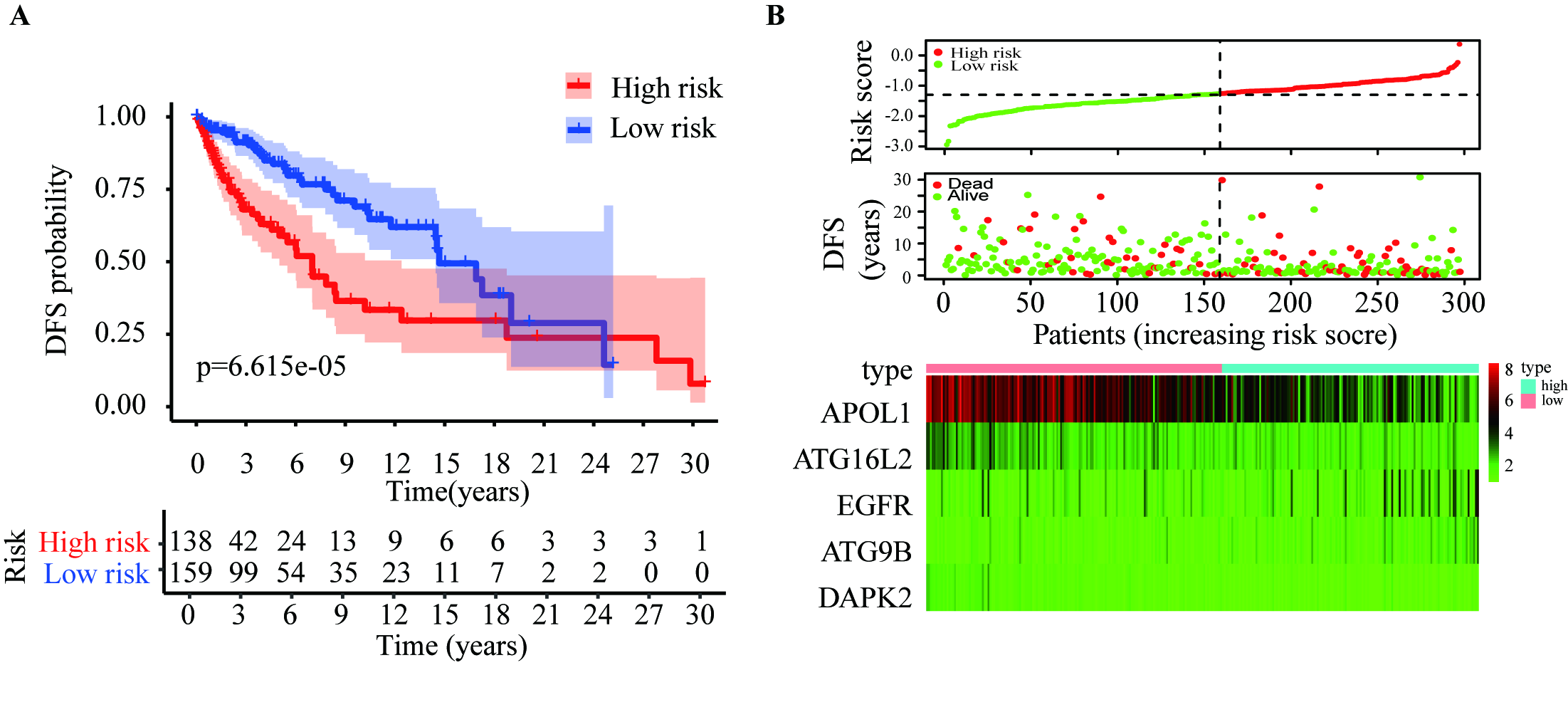

Supplement: Supplementary file 1 — Additional file 1: Figure S1. Disease free survival between high and low risk group. [file 12885_2021_8928_MOESM1_ESM.tif]

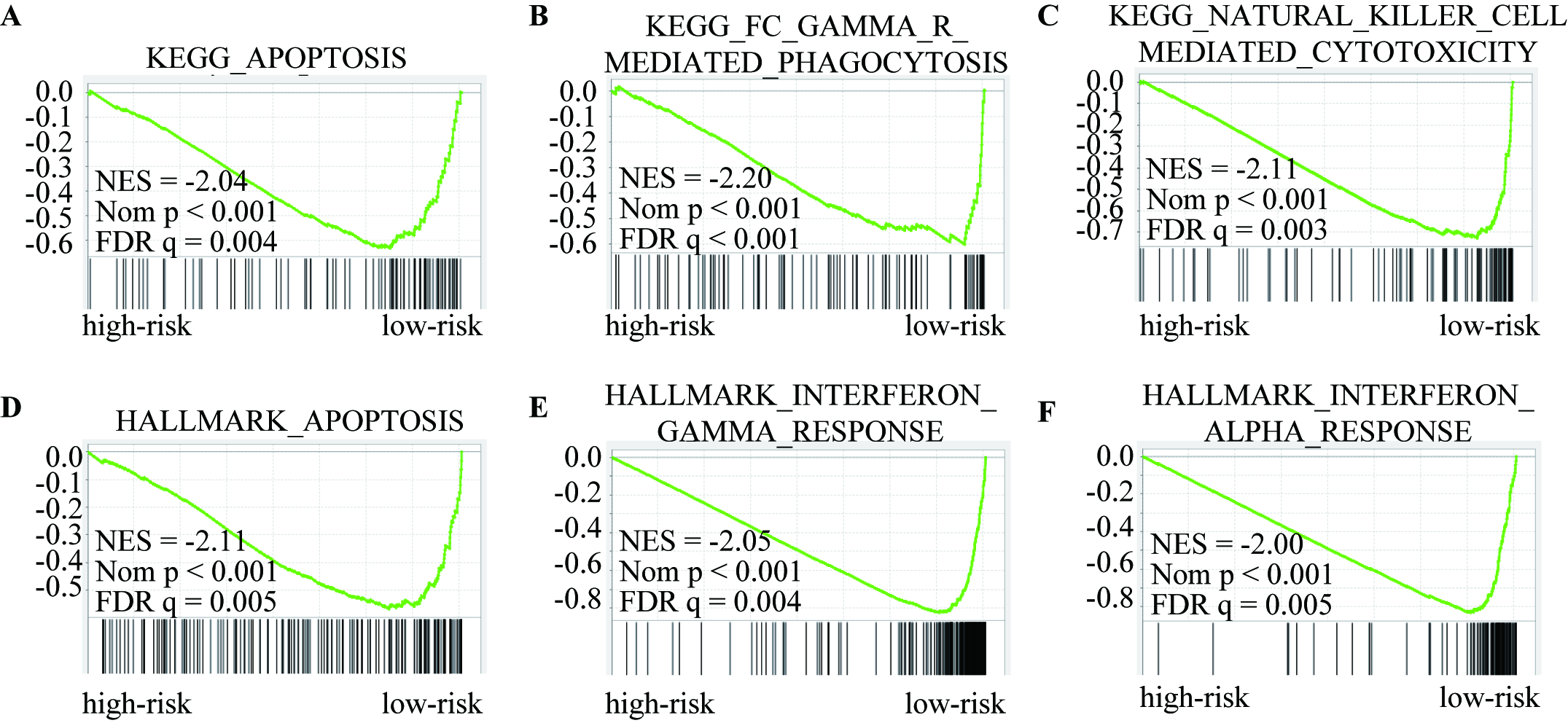

Supplement: Supplementary file 2 — Additional file 2: Figure S2. Validation of a prognostic nomogram for disease free survival. [file 12885_2021_8928_MOESM2_ESM.tif]

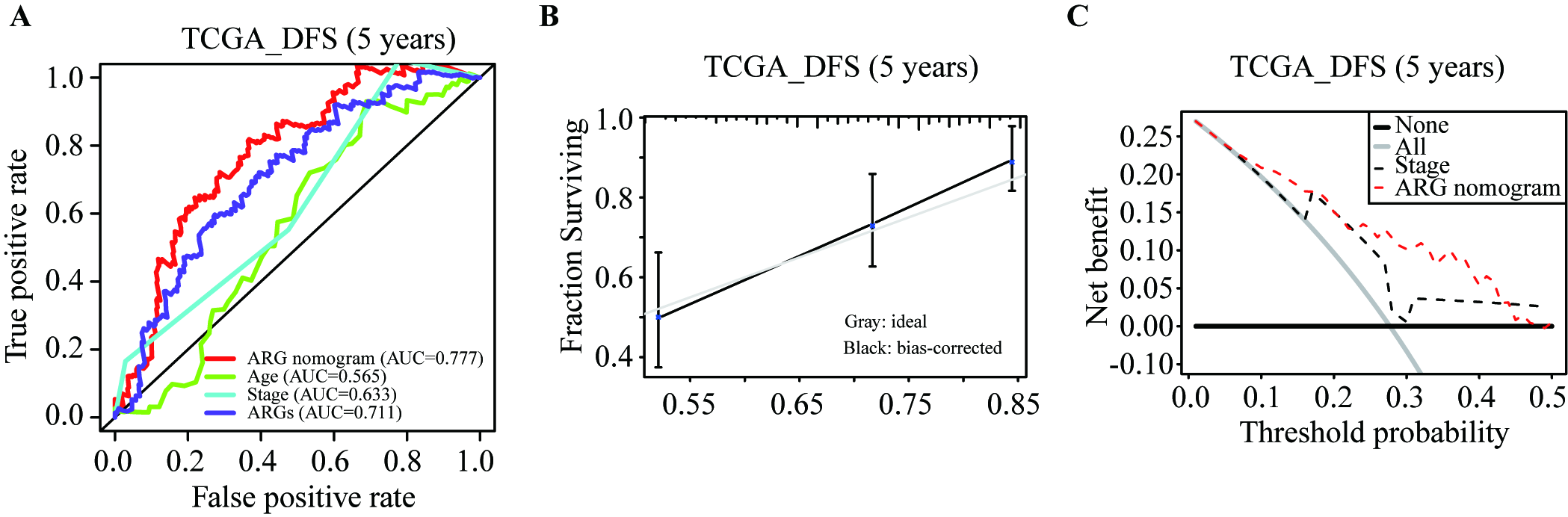

Supplement: Supplementary file 3 — Additional file 3: Figure S3. GSEA analysis between high and low risk group in KEGG (www.kegg.jp/kegg/kegg1.html) and HALLMARK gene sets. [file 12885_2021_8928_MOESM3_ESM.tif]

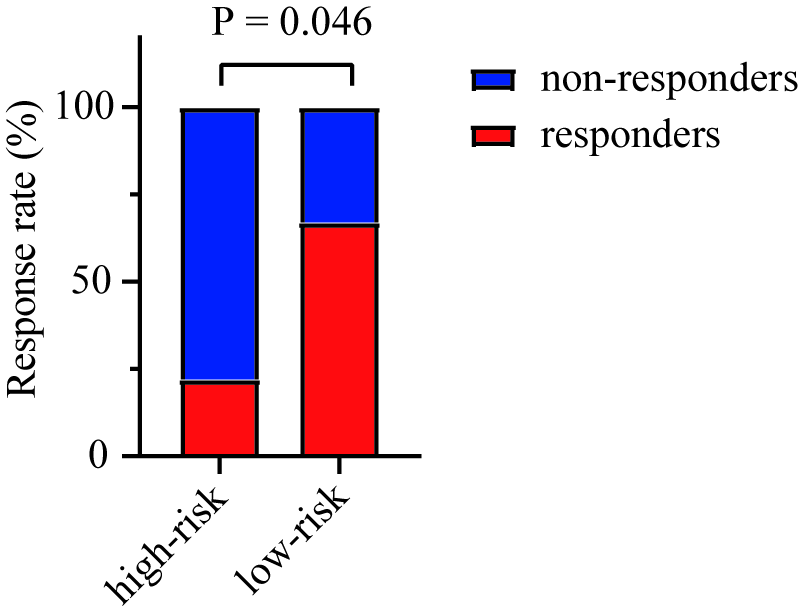

Supplement: Supplementary file 4 — Additional file 4: Figure S4. Immunotherapy response in low- and high-risk patients by ARGs signature. [file 12885_2021_8928_MOESM4_ESM.tif]
