# Supplementary material for: Nomogram based on autophagy related genes for predicting the survival in melanoma
Source: BMC Cancer. 2021 Nov 22;21:1258. doi: 10.1186/s12885-021-08928-9 (PMC8607622; doi:10.1186/s12885-021-08928-9)
Supplement: Supplementary file 5 — Additional file 5: Table S1. Clinical characteristics in TCGA and GEO datasets. [file 12885_2021_8928_MOESM5_ESM.docx]

**Supplementary table 1. Clinical characteristics in TCGA and GEO datasets.**

|  | TCGA | GTEx | GSE15605 | GSE46517 | GSE54467 |
| --- | --- | --- | --- | --- | --- |
| Total patients | 460 | - | 46 | 31 | 79 |
| Gender (n, %) |  |  |  |  |  |
| Male | 286 (62.3) | - | 32 | - | 50 (63.3) |
| Female | 174 (37.7) | - | 14 | - | 29 (36.7) |
| Age (mean ± SD, yr) | 58.1 ± 15.7 | - | 59.3 ± 16.6 | - | 56.2 ± 15.1 |
| Stage (n, %) |  |  |  |  |  |
| 0-I | 83 (18.1) | - | - | - | 29 (36.7) |
| II | 139 (30.2) | - | - | - | 29 (36.7) |
| III | 169 (36.7) | - | - | - | 20 (25.3) |
| IV | 23 (5.0) | - | - | - | 0 (0) |
| Unkown | 46 (10.0) | - | - | - | 0 (0) |
| OS (mean ± SD, m) | 61.4 ± 64.5 | - | - | - | 97.9 ± 47.3 |
| DFS | 57.4 ± 65.2 | - | - | - | - |
| Normal sample | 0 | 323 | 16 | 7 | 0 |

OS, overall survival; DFS, disease-free survival; yr, years; m, month.
